# Intraoperative Myelography in Transpsoas Lateral Lumbar Interbody Fusion for Degenerative Lumbar Spinal Stenosis: A Preliminary Prospective Study

**DOI:** 10.1155/2017/3742182

**Published:** 2017-11-02

**Authors:** Yang Yang, Liangming Zhang, Jianwen Dong, Zihao Chen, Peigen Xie, Ruiqiang Chen, Lei He, Feng Feng, Limin Rong, Bin Liu

**Affiliations:** Department of Spine Surgery, The Third Affiliated Hospital of Sun Yat-sen University, 600 Tianhe Road, Tianhe District, Guangzhou, Guangdong Province, China

## Abstract

**Aim:**

To investigate the feasibility and effectiveness of intraoperative myelography in determining adequacy of indirect spinal canal decompression during transpsoas lateral lumbar interbody fusion (LLIF).

**Methods:**

Seven patients diagnosed with degenerative lumbar spinal stenosis (DLSS) were prospectively included to this study. All patients underwent LLIF and subsequently received intraoperative myelography to determine the effect of indirect spinal canal decompression, which was visualized in both anterior-posterior and lateral images. Those patients with insufficient indirect canal decompression were further resolved by microendoscopic canal decompression (MECD). Radiological parameters, including stenosis ratio and dural sac area of operated levels, were measured and compared before and after operation. Besides, all patients were followed up for at least one year using visual analogue scale (VAS) for back and leg, Japanese Orthopaedic Association score (JOA), and Oswestry disability index (ODI).

**Results:**

Seven patients with 8 operated levels underwent LLIF safely and demonstrated significant symptom relief postoperatively. Five operated levels showed adequate indirect canal decompression intraoperatively, while the remaining three levels did not achieve the adequacy, and their residual stenosis was resolved following MECD. Radiological parameters were improved statistically when compared with preoperation (*P* < 0.05). Furthermore, neurological symptoms of all patients were also improved significantly (*P* < 0.05), shown by improved VAS (back and leg), JOA, and ODI at both two-week and one-year follow-up.

**Conclusions:**

Intraoperative myelography during LLIF is able to assess adequacy of indirect canal decompression for DLSS, thus promising favorable clinical outcomes.

## 1. Introduction

Degenerative lumbar spinal stenosis (DLSS) is characterized by disc protrusion with thickening of ligamentum flavum and hypertrophy of facet joint, at times, combined with lumbar spondylolisthesis or instability [1, 2]. When conservative therapies fail, traditional surgeries from posterior approach are widely used, including microendoscopic decompressive laminotomy for simple DLSS, posterior lumbar interbody fusion, transforaminal lumbar interbody fusion, and minimally invasive transforaminal lumbar interbody fusion for DLSS with lumbar spondylolisthesis or instability. However, these techniques can cause different levels of posterior ligament and muscle injury [2–5].

Nowadays, lateral route for canal decompression and interbody fusion named transpsoas lateral interbody fusion (LLIF) is gaining more popularity, and most procedures can be performed through a single shortened operative incision [6]. Compared with posterior surgeries, a larger interbody cage can be implanted, thus achieving more indirect decompression of spinal canal through further increase of foramina and canal area, as well as disc height. Advantages of LLIF using lateral transpsoas approach also include minimized damage of posterior anatomical structures, decreased intraoperative blood loss, shortened hospitalization, reduced postoperative pain, and enhanced fusion rate [7–9]. For posterior surgeries, adequacy of canal decompression can be assessed by direct visualization or probing; however, with regard to LLIF, it may be difficult to be estimated due to incompetence of visual or tactile feedback caused by confined surgical field and limited working channel. In order to ensure sufficient canal decompression to achieve desired clinical outcomes, we use a relatively simple and inexpensive protocol which incorporates intraoperative myelography into surgical manipulations. Aim of this preliminary prospective study is to illustrate feasibility and effectiveness of intraoperative myelography in LLIF for DLSS.

## 2. Patients and Methods

### 2.1. General Data

During recent two and a half years, seven patients (five males and two females) were included in this study. Their average age was 63 years, while mean duration of their neurological symptoms was approximately 6 years. A total of eight levels were to be operated: two in L2-3, three in L3-4, and three in L4-5 (Table 1). Their surgical inclusion criteria were mild to moderate lateral recess or foramina stenosis induced by thickening ligamentum flavum, combined with image-confirmed segmental instability (Posner criteria, five patients) or spondylolisthesis (Meyerding Grade I, two patients) but without obvious facet hypertrophy (Fujiwara Grade I or Grade II) [10, 11]. Their mechanical back pain and neurological symptom could not be relieved by conservative medications for at least six weeks. However, patients revealing obvious central canal stenosis, congenital canal stenosis, locked facets, spondylolisthesis (Meyerding Grade II or more), moderate to severe scoliosis, or mere segmental canal stenosis without radiographic evidence of instability or spondylolisthesis were ruled out in this research. All of them underwent preoperative myelography and were operated on by one senior surgeon in our division. This study was approved by institutional ethic committee of the hospital and informed consent of the whole surgery, including possible direct canal decompression, was obtained from each participant.

### 2.2. Procedures of LLIF

Under general anesthesia, patient was flexed in a standard 90° lateral decubitus position with the left side elevated and taped in this position. A bump or roll was put under the right side to further increase distance between iliac crest and rib cage. After sterilizing skin and draping, one single lateral incision with the length of about 3 cm was made to insert probe exactly over the pathological level under fluoroscopy. Then sequential dilators were placed accurately through the same approach. It must be ensured that psoas was parted between the middle and anterior third of the muscle, guaranteeing that lumbar plexus was posteriorly located and outside operative corridor. Blunt dissection of psoas muscle was achieved by expanding dilator blades to expose lateral margin of operated disc. A thorough discectomy was performed through lateral route under direct vision until contralateral annulus could be observed, however, leaving posterior annulus intact. Following well preparation of end plates, a proper cage inserted with autogenous bone, which was harvested from ipsilateral iliac crest, was positioned on bilateral margins of the epiphyseal ring. After irrigation and closure of wound, intraoperative myelography was performed in lateral position.

### 2.3. Intraoperative Myelography and Microendoscopic Canal Decompression

Interspinous space of surgical level was identified and 15 ml nonionic water-soluble iohexol medium was injected into subarachnoid space via dorsal approach by using 22 G-23 G spinal puncture needle. After prone positioning, both anterior-posterior and lateral myelogram were obtained by G-arm portable X-ray machine to compare with preoperative images. Any remaining canal stenosis or lateral recess impingement was identified as filling defect in myelogram. Besides clinical experiences, the surgeon mainly determined adequacy of canal indirect decompression based on lateral stenosis ratio (SR) of more than 70% at surgical level. Microendoscopic canal decompression (MECD) was performed for those who still revealed remaining stenosis following lateral cage implantation. A transverse incision targeted for operated level was made under fluoroscopic guidance. After incising fascia and detaching paraspinal muscles, serial tubular dilators were used to well position the working canal; afterwards endoscopic system was mounted onto the working canal. Thorough canal decompression was performed under microendoscopy (Medtronic METRx system) and its adequacy was demonstrated by real-time video images, as well as another myelogram immediately after canal direct decompression. Finally, for all patients, posterior pedicle screws and rods were inserted and tightened percutaneously under fluoroscopic guidance from G-arm portable X-ray machine (one case for unilateral fixation and six cases for bilateral implantation).

### 2.4. Clinical Assessments and Statistical Analysis

**Figure 1:**
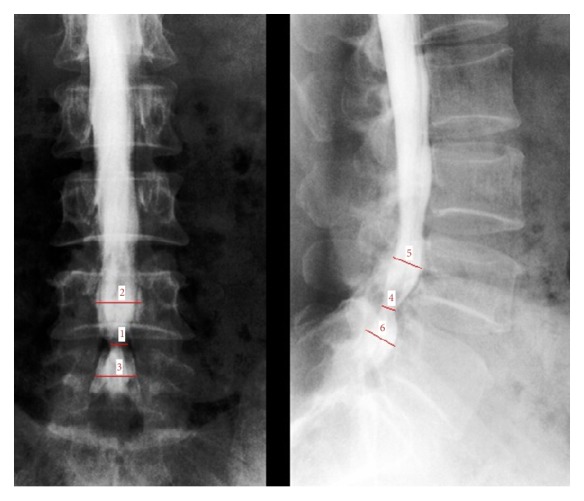
*Measurement of stenosis ratio (SR) at L4-5 level*: Line 1/[(Line 2 + Line 3)/2] × 100% (anterior-posterior position), Line 4/[(Line 5 + Line 6)/2] × 100% (lateral position).

In order to quantify severity of canal stenosis in plain myelogram, SR was defined as ratio of dural sac width in pathological level upon the arithmetic mean of that at upper and lower middle vertebrae. For instance, SR in L4-5 level was calculated as follows: (dual sac width at L4-5 level/dural sac width averaged at L4 and L5 middle vertebrae levels) × 100% (Figure 1). In this study, SR preoperation (several days before surgery) and postoperation (after lateral interbody cage implantation and possible MECD) in both anterior-posterior and lateral myelogram were calculated. Based on transverse computed tomography (CT) image, both preoperative and postoperative dural sac area of operated levels (one day after surgery) were measured. All radiological measurements were performed by three case-blinded assessors, and their mean values were used in this research. Meanwhile, clinical outcomes before operation and after operation (two weeks and one year following surgery), including visual analogue scale (VAS) for back and leg, Japanese Orthopaedic Association score (JOA), and Oswestry disability index (ODI), were also evaluated. All these parameters were compared using paired *t*-test, and statistical significance was defined as *P* < 0.05 in this study.

## 3. Results

**Figure 2:**
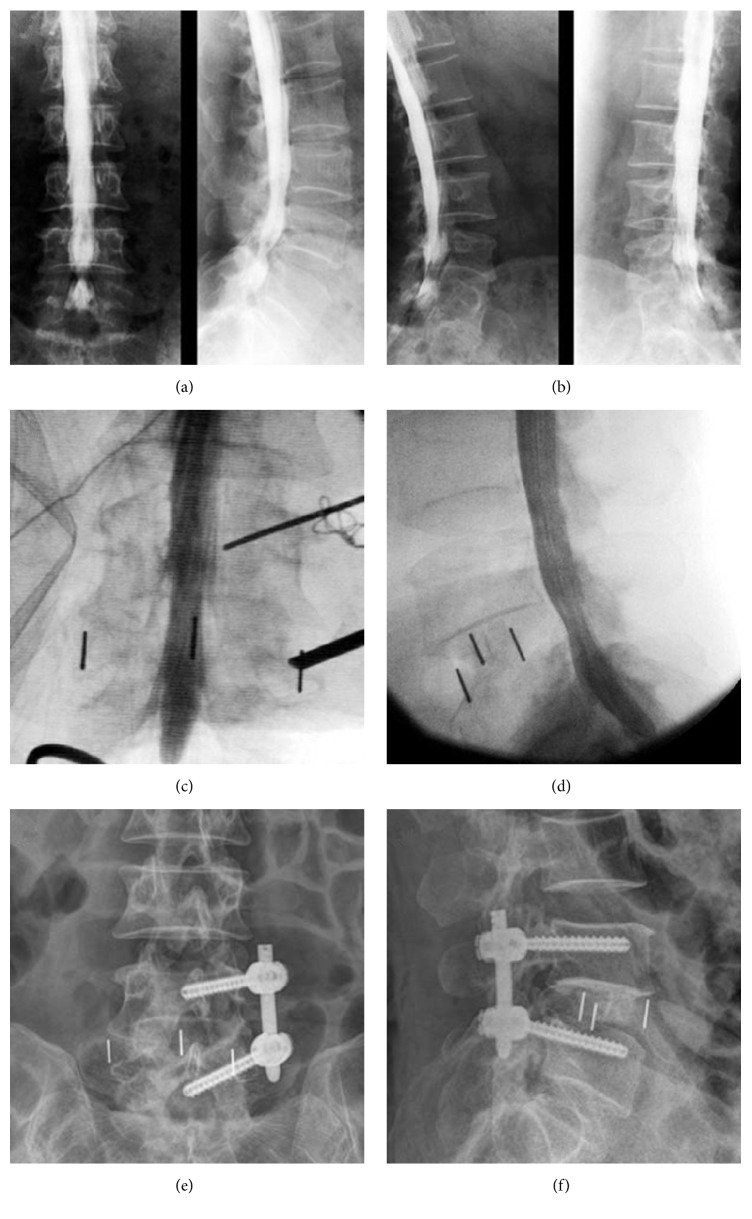
*Radiographs of Case (B)*: preoperative myelography indicated canal stenosis of L4-5 level ((a) and (b)). Intraoperative myelography during LLIF demonstrated resolution of filling defect ((c) and (d)). Postoperative lumbar plane images showed disc height increase at L4-5 level ((e) and (f)).

**Figure 3:**
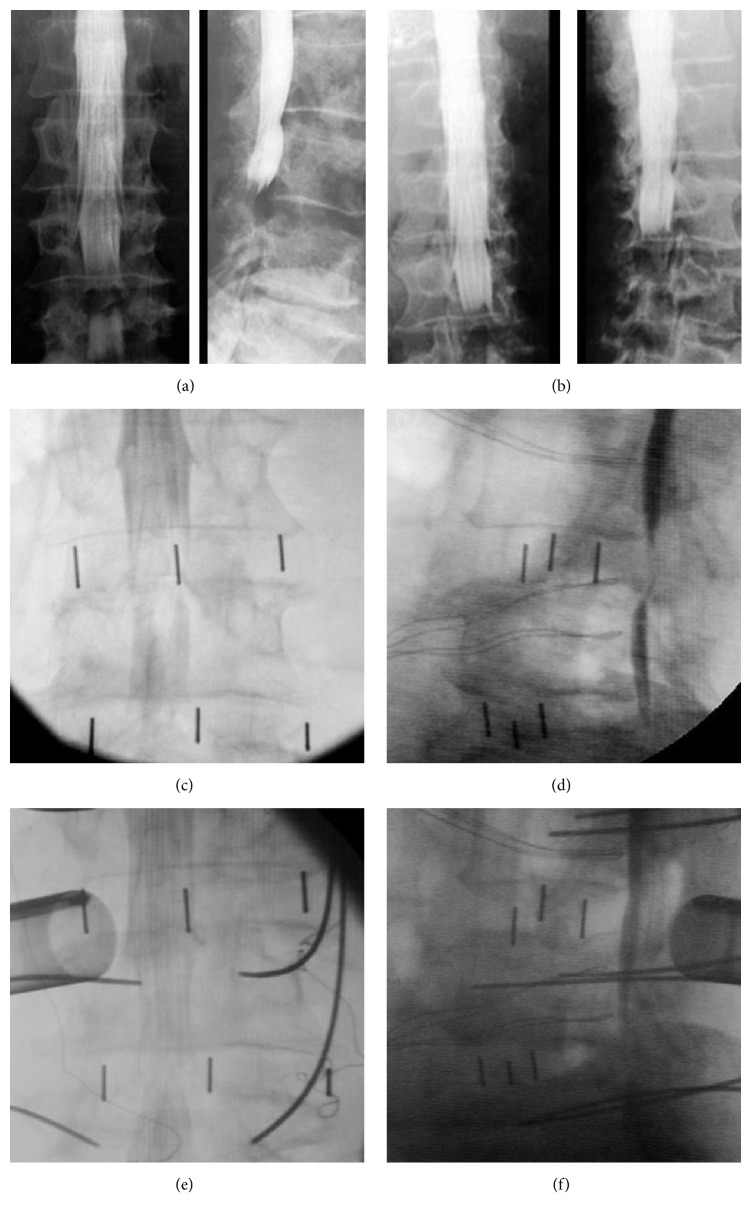
*Radiographs of Case (C)*: preoperative myelography identified filling defects of both L3-4 and L4-5 levels ((a) and (b)). Intraoperative myelography did not reveal adequate indirect canal decompression at L3-4 level ((c) and (d)). Sufficient canal decompression was achieved after same-level MECD ((e) and (f)).

All seven patients underwent LLIF successfully without perioperative complication. Operative duration and blood loss were approximately 60 minutes and 70 ml per level, respectively. Intraoperative myelogram of five operated levels demonstrated adequate indirect canal decompression (Figure 2), whereas myelogram of three remaining levels revealed residual stenosis caused by inadequate canal decompression. Subsequent same-level MECD was performed, and then another myelogram following MECD demonstrated no remaining filling defect (Figure 3). After the whole surgery (LLIF of five levels and additional MECD of remaining three levels), both anterior-posterior and lateral SR were statistically increased when compared with preoperation (both *P* < 0.05, Table 2). Based on transverse CT images, postoperative dural sac area of surgical levels was also enlarged significantly in comparison with preoperative one (*P* < 0.05, Table 2). Following operations, all seven patients showed obvious improvement of pain and neurological symptoms. Scores at both two weeks and one year postoperatively, including VAS (back and leg), JOA, and ODI, demonstrated significant improvement when compared with preoperation (all *P* < 0.05, Table 3). Disc heights, interbody dimensions, surgical indicators, and radiological measurements of all operated levels were listed in Supplementary Tables  1 and 2, while clinical assessments of each patient were showed in Supplementary Table  3, in Supplementary Material, available online at https://doi.org/10.1155/2017/3742182.

## 4. Discussion

Minimally invasive spinal surgeries have been gaining extensive popularity recently because of decreased iatrogenic trauma, comparable clinical outcomes, and shortened hospitalization [12–14]. For both open and minimally invasive operations, adequate canal decompression is still the key surgical goal [15]. With respect to LLIF, after placement of a large fusion cage by lateral route, disc height, canal, and foraminal area of surgical segment can increase greatly when compared with preoperation, and supplementary posterior pedicle screw instrumentation is able to afford a greater increase in disc height and foraminal area [9, 16, 17]. Thus, LLIF owns the effect of indirect decompression for DLSS. In comparison with direct decompression, it is less invasive and mainly determined on restoration of thickening ligamentum flavum. However, preoperative radiological images are not able to evaluate whether it would be restored, so how to estimate adequacy of indirect decompression still troubles surgeons, especially when intraoperative CT or magnetic resonance imaging (MRI) is not available. As preoperative myelography is more reliable and reproducible to identify responsible level in lumbar spinal stenosis for the decision of surgery [18, 19], it may be expected that intraoperative myelography is able to detect remaining canal stenosis following interbody cage implantation by lateral access [20].

In this study, for five operated levels requiring no additional MECD, it indicates that thickening ligamentum flavum is buckled and can be restored following cage implantation via lateral route, thus promising favorable indirect decompression effect, while for three remaining levels thickening ligamentum flavum can not be restored effectively possibly due to its excessive hypertrophy or inserted cage with inappropriate height, so that limited indirect decompression does not guarantee adequacy of canal decompression. Therefore, MECD is performed to ensure that goal. Postoperatively, all seven patients report satisfying relief of neurological symptoms, proving feasibility of intraoperative myelography in determining the extent of indirect decompression for LLIF. Besides, intraoperative myelography provides precise localization for residual canal stenosis during surgery, so that surgeon can identify filling defect in myelogram as decompression field and further evaluate severity of external compression. It may also avoid potential location fault of operated level by only using anatomic landmark under fluoroscopic guidance, intraoperative unnecessary soft tissue detachment and bony resection. This study introduces SR as one quantitative indicator to evaluate the extent of canal stenosis. With the increase of its value, the stenosis severity gradually decreases. Postoperative anterior-posterior and lateral SR of five levels with no additional MECD are significantly increased when compared with preoperation, adding new proof to the effect of indirect decompression for LLIF, while increase of SR for the remaining three levels after additional MECD also indirectly proves the efficacy of intraoperative myelography in discovering residual canal stenosis.

Recent studies have found that severity of lumbar spinal stenosis can change with different body positions. Specifically, forward flexion and relaxed supine position provide larger spinal canal volume, thus relieving neurological symptom. While extension position can decrease its volume and reduce dural sac cross-sectional area [21–24]. Preoperative images of CT or MRI are often acquired in relaxed supine position, so it may underestimate severity of lumbar canal stenosis to some extent. Intraoperative myelogram at different positions, such as flexion or extension, can be easily obtained, so the surgeon is able to assess the severity of lumbar canal stenosis more accurately. Based on these, it is considered as one crucial supplemental method to confirm external impingement and may not be replaced by CT or MRI totally at present [18, 25, 26]. Other advantages of intraoperative myelography over CT or MRI include easy manipulation and inexpensiveness [27, 28]. For patients with multilevel stenosis, severe segmental stenosis, and structural abnormalities or needing revision operations, intraoperative myelography is especially recommended to ensure adequate canal decompression [3, 27]. However, some potential risks of intraoperative myelography should draw surgeon's attention, including meningitis because of improper skin disinfection, headache induced by cerebrospinal fluid leak, subarachnoid hematoma, and nerve injury due to inappropriate manipulation, as well as rare anaphylactic reaction-seizure caused by contrast medium. Careful preoperative preparation and normative intraoperative performance are the best prophylactic measures [29–31].

Some limitations need to be resolved before gaining more acceptance of intraoperative myelography for LLIF. First, larger sample size should be acquired and control group receiving no intraoperative myelography also needs to be established to verify efficacy of SR in determining resolution of canal stenosis. Besides, longer-term follow-up is necessary to further confirm clinical outcomes of LLIF.

## 5. Conclusion

Intraoperative myelography is considered to be able to identify remaining stenosis even if in absence of real-time CT and MRI, thus confirming adequacy of indirect canal decompression during LLIF.

## Supplementary Material

Supplementary Table 1 shows disc height before and after surgery and its change, as well as implanted interbody cage dimensions. Supplementary Table 2 reveals comparisons of surgical and radiological measurements between requiring no MECD levels and requiring MECD levels. Supplementary Table 3 shows comparisons of postoperative clinical assessments at different time intervals between requiring no MECD cases and requiring MECD cases.

## Figures and Tables

**Table 1:** General data of operated cases.

Case	Gender	Age (years)	Main symptom	Disease course (year)	Operated level	Follow-up (months)
(A)	Male	71	Neurological intermittent claudication and Back pain	2	L2-3	12
(B)	Female	45	Neurological intermittent claudication and back pain	10	L4-5	30
(C)	Male	62	Neurological intermittent claudication and back pain	10	L3-4, L4-5	20
(D)	Male	83	Neurological intermittent claudication and back pain	3	L2-3	16
(E)	Male	59	Neurological intermittent claudication and back pain	5	L4-5	18
(F)	Male	65	Neurological intermittent claudication	3	L3-4	24
(G)	Female	56	Neurological intermittent claudication and back pain	10	L3-4	14

**Table 2:** Radiological measurements before operation and after operation.

	Before operation	After operation	*P* value
SR (anterior-posterior)	(26.1 ± 24.1)%	(84.2 ± 12.1)%	0.000
SR (lateral)	(33.1 ± 31.8)%	(81.1 ± 12.9)%	0.005
Transverse dural sac area (cm^2^)	0.65 ± 0.22	1.41 ± 0.39	0.000

SR: stenosis ratio.

**Table 3:** Clinical assessments before operation and after operation.

	Before operation	Postoperative two weeks	Postoperative one year	*P* value^*※*^
VAS (back)	5.0 ± 1.2	1.9 ± 0.6	1.7 ± 0.7	0.000
VAS (leg)	6.1 ± 0.9	1.7 ± 0.9	1.6 ± 0.7	0.000
JOA	14.7 ± 2.1	24.9 ± 1.0	25.1 ± 1.1	0.000
ODI	(50.6 ± 4.9)%	(31.7 ± 3.8)%	(28.9 ± 2.8)%	0.000

VAS: visual analogue scale; JOA: Japanese Orthopaedic Association score; ODI: Oswestry disability index. ^*※*^Compared with preoperation.
