# Supplementary material for: Intraoperative Myelography in Transpsoas Lateral Lumbar Interbody Fusion for Degenerative Lumbar Spinal Stenosis: A Preliminary Prospective Study
Source: Biomed Res Int. 2017 Nov 2;2017:3742182. doi: 10.1155/2017/3742182 (PMC5688347; doi:10.1155/2017/3742182)
Supplement: Supplementary file 1 — Supplementary Table 1 shows disc height before and after surgery and its change, as well as implanted interbody cage dimensions. Supplementary Table 2 reveals comparisons of surgical and radiological measurements between requiring no MECD levels and requiring MECD levels. Supplementary Table 3 shows comparisons of postoperative clinical assessments at different time intervals between requiring no MECD cases and requiring MECD cases. [file 3742182.f1.docx]

**Supplementary Table 1. Disc Height and Interbody Cage of Operated Levels**

| **Level Preoperative disc height (mm) Postoperative disc height (mm) Disc height improvement Interbody cage (mm,** °**)**  Anterior Middle Posterior Anterior Middle Posterior Anterior Middle Posterior Width Length Height Lordosis angle |
| --- |
| **L2-3** 7.4 6.0 2.8 8.0 7.7 3.1 8.1% 28.3% 10.7% 18 52 10 6  **L4-5** 7.0 8.0 2.8 15.2 12.4 4.2 117.1% 55.0% 50.0% 18 52 10 6  **L3-4** 10.1 10.1 4.2 11.4 15.6 5.1 12.9% 54.5% 21.4% 18 52 12 6  **L4-5** 17.2 11.3 6.0 22.0 14.1 10.8 27.9% 24.8% 80.0% 18 50 13 6  **L2-3** 17.2 12.0 7.6 20.4 19.0 13.4 18.6% 58.3% 76.3% 18 50 12 6  **L4-5** 7.8 8.3 5.9 19.5 14.6 10.8 150.0% 75.9% 93.1% 18 50 13 6  **L3-4** 12.3 11.9 8.1 14.9 16.0 11.2 21.1% 34.5% 38.3% 18 50 11 6  **L3-4** 8.7 6.8 5.6 12.3 13.4 8.3 41.4% 97.1% 48.2% 18 45 13 6 |

**Supplementary Table 2. Surgical and Radiological Measurements**

**Between Requiring no MECD Levels and Requiring MECD Levels**

| **Requiring no MECD (five levels) Requiring MECD (three levels)** |
| --- |
| **Blood loss** (ml) 55.2 ± 2.1 68.0 ± 2.2  **Surgical duration** (min) 62.0 ± 3.0 83.3 ± 1.2  **Intraoperative SR** (anterior-posterior) (84.6 ± 12.2)% (41.1 ± 1.6)% *  **Intraoperative SR** (lateral) (80.0 ± 12.7)% (49.1 ± 2.1)% *  **Postoperative transverse dural sac area** (cm^2^) 1.37 ± 0.34 1.47 ± 0.45 |

**SR** stenosis ratio

* The first measurement after intraoperative myelography (before MECD)

**Supplementary Table 3. Postoperative Clinical Assessments**

**Between Requiring no MECD Cases and Requiring MECD Cases**

| **Requiring no MECD (four cases) Requiring MECD (three cases)**  two weeks one year two weeks one year |
| --- |
| **VAS** (back) 1.8 ± 0.8 1.8 ± 0.8 2.3 ± 0.5 2.0 ± 0.8  **VAS** (leg) 1.8 ± 0.4 1.3 ± 0.4 1.7 ± 1.2 2.0 ± 0.8  **JOA** 24.5 ± 0.5 24.8 ± 0.8 25.3 ± 1.2 25.7 ± 1.2  **ODI** (32.0 ± 3.5)% (28.0 ± 2.4)% (31.3 ± 4.1)% (30.0 ± 2.8)% |

**VAS** Visual analogue scale

**JOA** Japanese Orthopaedic Association score

**ODI** Oswestry disability index
